# Parliament and the Professions

**Published:** 1920-11-27

**Authors:** 


### Parliament and the Professions.

### Maternity and Child Welfare.

The Minister of Health, in answer to a question, stated that the following local authorities have made arrangements under Section 1 of the Maternity and Child Welfare Act, 1918: The whole of the twenty-nine Metropolitan Borough Councils (including the Common Council of the City of London); of the eighty-two County Borough Councils in England and Wales; of the sixty-two County Councils in England and Wales (except the London County Council, which has no powers under the section, and the Glamorganshire County Council), and the Councils of 306 of the 1,703 sanitary districts in England and Wales, including most of the larger non-county boroughs and urban districts. Of the remaining 1,397 sanitary districts, 1,390 are included in County Councils' schemes, and are covered by arrangements made under such schemes. The seven districts in which no arrangements have been or are being made are small urban and rural districts.

### Medical Insurance Service.

Dr. Addison stated that the four divisional medical officers appointed in connection with the new medical insurance service are Dr. A. Fulton, M.B., North-Western Division; Dr. C. H. Milburn, M.D., North-Eastern Division; Dr. H. J. Neilson, M.D., Southern Division; and Dr. R. E. Crosse, M.R.C.S., Metropolitan and Eastern Division. They all receive £1,600 a year net inclusive salary.

### The Government and Voluntary Hospitals.

Dr. Addison informed Sir F. Hall that he was aware of the report as to the closing of the London Hospital, with regard to the special difficulties of which he hoped the National Relief Fund would assist through their grant of £700,000 towards war deficits of the voluntary hospitals in the United Kingdom. He stated that he was anxious to preserve the voluntary system, and the proposals of the Government as contained in Clause 11 of the Ministry of Health (Miscellaneous Provisions) Bill are put forward with a view to assisting the present urgent necessity.

### Epidemics in London.

Dr. Addison stated that scarlet fever and diphtheria have been exceptionally prevalent in London this autumn, but both diseases are of an extremely mild type, and the death rate in each case is far below that experienced in previous epidemics. There is no evidence of the existence of epidemic influenza in the country at the present time. The origin of these waves of zymotic disease is obscure. Similar, although more fatal, outbreaks occurred in 1892 and 1893. The existing machinery and the available hospital accommodation have proved equal to the task of coping with the present outbreaks.

